# Evaluation of drug provocation tests without prior skin testing in children with suspected penicillin allergy and correlation with PEN-FAST: A single-center study

**DOI:** 10.1007/s00431-025-06301-7

**Published:** 2025-07-17

**Authors:** Halime Yağmur, Özge Atay, Damla Baysal Bakır, Gizem Kabadayı, Özge Kangallı Boyacıoğlu, Suna Asi̇lsoy, Nevin Uzuner

**Affiliations:** 1 Department of Pediatric Immunology and Allergy, Dokuz Eylul University, İzmir, Türkiye; 2 Department of Pediatric Immunology and Allergy, Çiğli Training and Research Hospital, Bakırçay University, İzmir, Türkiye; 3 Allergos Allergy Clinic, İzmir, Türkiye

**Keywords:** Penicillin, Drug provocation test, Beta-lactam allergy, PEN-FAST, Children, Safety

## Abstract

**Purpose:**

To evaluate patients with suspected penicillin allergy in whom skin testing was omitted during immediate and delayed reactions before drug provocation test (DPT).

**Methods:**

This retrospective study analyzed patients aged 0–18 years with suspected penicillin allergy between 2020 and 2023. Data on hypersensitivity reaction history, laboratory tests, PEN-FAST scores, and DPT records were collected.

**Results:**

We evaluated 75 patients (male: 61.3%; median age at index reaction: 4 [range: 1–15] years) with suspected penicillin allergy. Nearly all reactions occurred at home (98.7%) following oral administration, with antihistamines being the most common treatment (56%). Urticaria was the most frequent manifestation in immediate reactions (30.7%), whereas maculopapular exanthema was predominant in delayed reactions (33.7%). Amoxicillin-clavulanic acid was the most frequently implicated drug (85.3%). The median PEN-FAST score was 3 (range: 0–5). Immediate reactions were significantly more common in females (*p* < 0.05). In total, 78 DPTs were performed, and four patients tested positive, all experiencing mild cutaneous reactions. No life-threatening reactions were observed. At the 3-month follow-up, 90.7% of patients tolerated beta-lactam antibiotics, though 4% chose to avoid them despite negative DPT results.

**Conclusion:**

In our study, we successfully performed DPT without major complications by omitting skin testing in both immediate and delayed reactions. Our findings suggest that in delayed reactions, direct DPT can safely replace skin testing. Notably, we also propose that after in vitro tests, DPT may be safely used in immediate reactions without additional in vivo testing. Also, this is the first study to evaluate PEN-FAST in children with suspected penicillin allergy in our country, offering practical value for clinicians and patients.

**Table:** 

**What is Known:** • *The label of drug allergy in children is assigned far more frequently than the actual diagnosis of true allergic reactions.* • *Due to unverified drug allergy diagnoses, patients face challenges such as inadequate treatment and the development of antibiotic resistance.*
**What is New:** • *In cases with immediate reactions, including anaphylaxis, direct drug provocation test (DPT) performed without prior skin testing can be conducted without severe reactions.* • *The PEN-FAST score has been observed to be highly effective in ruling out beta-lactam allergy in pediatric patients with low scores.*

## Introduction

Drug hypersensitivity reactions are adverse drug reactions that are generally unpredictable and may be mediated by both immune and nonimmune mechanisms [1]. The reported prevalence of drug allergy in children ranges from 2.8% to 7.8% [2, 3]. Beta-lactam (BL) antibiotics are the most frequently implicated agents in these reactions [4]. However, confirmed beta-lactam allergy (BLA) is not as common as often perceived [5, 6]. Because of an unconfirmed drug allergy label, patients are frequently prescribed alternative antibiotic classes, which may lead to treatment failures, the development of antibiotic resistance, and increased healthcare costs [7, 8]. Therefore, an accurate diagnosis of drug allergy is necessary [9].

Suspected reactions are classified as immediate or delayed based on the timing of drug intake relative to the onset of symptoms [7, 8]. In the clinical evaluation of suspected penicillin allergy, the PEN-FAST score—incorporating factors such as a history of penicillin allergy, a reaction occurring within 5 years, the presence of anaphylaxis or angioedema, severe cutaneous/systemic reactions, and the treatment administered—can be used for rapid risk stratification [10]. After obtaining a detailed history from specialists, an appropriate in vivo and/or in vitro testing strategy should be devised according to the type of reaction. Drug provocation test (DPT) is considered the gold standard for diagnosing drug allergy [11, 12].

In the literature, for BL allergies in children with a history of immediate reactions, it is still recommended to perform skin tests followed by a graded DPT. Skin testing remains valuable and informative, particularly in cases with a history suggestive of immediate reactions, as it helps prevent potentially severe responses. However, recent studies have presented alternative perspectives, reporting that in selected cases of immediate drug reactions, direct DPT may be performed without preceding diagnostic skin tests. [13, 14]. In delayed reactions, DPT is generally not recommended, except in patients with mild-to-moderate risk. In the present study, direct DPT was implemented in our cases due to the necessity of using alternative antibiotics, the risk of antibiotic resistance, and the limited availability of testing materials. We retrospectively evaluated pediatric patients who underwent DPT due to suspected penicillin/BL allergy.

## Materials and methods

### Population and specific IgE

Patients aged 0–18 years who presented with suspected BLA at the pediatric allergy and immunology clinic of our hospital between January 2020 and December 2023 were identified. For all patients, drug allergy records were reviewed, which included details on the spectrum and timing of symptoms, route of administration, history of previous hypersensitivity reactions, and physical examination findings. Patients who underwent routine penicillin V- and penicillin G-specific IgE antibody testing (CAP-FEIA, Thermo Fisher Scientific, Uppsala, Sweden) and those who did not undergo skin tests were identified [15]. A penicillin-specific IgE level ≥ 0.35 kUA/L was considered positive, whereas levels < 0.35 kUA/L were considered negative. Based on clinical history, reactions occurring within 1 h were classified as immediate and those occurring after 1 h were defined as delayed [16].

According to World Allergy Organization criteria, anaphylaxis was diagnosed when, following exposure to the suspected allergen, the patient exhibited either sudden onset of hypotension, bronchospasm, or laryngeal involvement, or abrupt cutaneous/mucosal manifestations accompanied by involvement of the respiratory system, hypotension, or persistent gastrointestinal symptoms [17]. Anaphylaxis was further classified by severity as mild, moderate, or severe according to the criteria of Brown et al.[18]. Patients diagnosed with severe anaphylaxis, life-threatening severe cutaneous systemic reactions (including acute generalized exanthematous pustulosis [AGEP], Stevens–Johnson syndrome/toxic epidermal necrolysis [SJS/TEN], drug reactions with eosinophilia and systemic symptoms [DRESS], or other organ-specific drug reactions), severe cardiovascular disease, severe pulmonary disease and patients in which informed parental consent for DPT was not obtained were excluded from the study.

For patients with suspected penicillin allergy, clinical risk was evaluated using the PEN-FAST scoring system. In this system, 2 points were assigned if the index reaction occurred within 5 years; 2 points for the presence of anaphylaxis, angioedema, or a severe cutaneous adverse reaction; and 1 point if treatment was required during the index reaction. Based on the total score, patients were classified as follows: 0 = very low risk, 1–2 = low risk, 3 = intermediate risk, and 4–5 = high risk [10].

Demographic data, clinical and laboratory findings (total IgE and eosinophil count), and DPT outcomes were recorded for patients with negative penicillin-specific IgE who subsequently underwent DPT.

### Drug provocation test

All DPTs were performed in our clinic, which is fully equipped with the necessary personnel, physicians, and emergency equipment to manage acute situations. Before performing DPT, each patient underwent a systemic examination, and physical findings were assessed both before and after the procedure. Any conditions or medications that could interfere with the test were avoided. Detailed information was provided to the families of all patients before DPT, and informed consent was obtained.

DPT was performed using the same drug implicated in the patient’s reaction [19], with the dosage calculated according to the patient’s age and weight and administered orally. On the first day of provocation, the daily therapeutic dose was divided into four doses that were logarithmically increased at 30-min intervals. Following the final dose, patients were placed under observation in the hospital for 4 h [20, 21]. The provocation test was considered positive if any changes in cutaneous findings; respiratory, cardiovascular, or gastrointestinal symptoms; or vital signs were observed during or after the test. For patients with a history of delayed reactions, the therapeutic daily dose was divided into two doses and administered at home over a 5-day period. Clinical findings for patients who developed reactions were documented. At least 3 months after the DPT, patients were queried regarding the occurrence of beta-lactam-related reactions.

### Ethical approval

Ethical approval for the study was obtained from the Local Ethics Committee of Dokuz Eylül University Faculty of Medicine (Decision No: 2024/17–08) and was performed following the ethical standards as laid down in the Declaration of Helsinki and its later amendments.

### Statistical analysis

Data analysis was performed using IBM SPSS Statistics Version 26 (IBM Corp., Armonk, NY, USA). The normality of the data distribution was determined using the Kolmogorov–Smirnov test. Descriptive statistics for patient demographics and laboratory parameters were presented as number (n), percentage (%), minimum value, maximum value, and median (interquartile range [IQR]).

Chi-square and Mann–Whitney *U* test were used for comparisons between drug reactions with respect to the subcategories of duration, sex, and family history of allergy. All results with a pvalue < 0.05 were considered statistically significant.

## Results

A total of 75 patients with suspected penicillin allergy were evaluated. The median age at the index reaction was 4 years (IQR: 4; range: 1–15 years), and 61.3% of the patients were male. All patients had used antibiotics for the treatment of an infection, and nearly all had taken the suspected drug orally (*n* = 74; 98.7%). The index reaction occurred predominantly in the home environment (*n* = 74; 98.7%), and antihistamines were the most frequently administered treatment after reaction (*n* = 42; 56%). In patients with immediate reactions, urticaria was the most common manifestation (30.7%), whereas maculopapular exanthema was most frequently observed in those with delayed reactions (33.7%). Amoxicillin-clavulanate was the most commonly implicated drug in these reactions (*n* = 65; 85.3%).

Laboratory evaluations revealed a median total IgE level of 56 IU/mL (range: 2.4–1894 IU/mL), a median eosinophil count of 100 mm3/L (range: 0–900 mm3/L), and a median eosinophil percentage of 1.6% (range: 0–7.3%). All patients had negative penicillin V- and penicillin G-specific IgE results. Allergic rhinitis was the most commonly identified comorbid allergic condition among the patients. According to parental allergy histories, among mothers, 72% (*n* = 54) had no concomitant atopic disease, 8% (*n* = 6) had a history of drug allergy, 8% (*n* = 6) had allergic rhinitis, 5.3% (*n* = 4) had asthma, 4% (*n* = 3) had chronic urticaria, 1.3% (*n* = 1) had atopic dermatitis, and 1.3% (*n* = 1) had contact dermatitis. Among fathers, 88% (*n* = 66) had no concomitant atopic disease, 8% (*n* = 6) had allergic rhinitis, 1.3% (*n* = 1) had a drug allergy, 1.3% (*n* = 1) had asthma, and 1.3% (*n* = 1) had chronic urticaria. Demographic data and clinical findings for the patients are summarized in Table 1.

**Table 1:** Demographic characteristics and clinical data of the patients

	n (%)
**Sex**	
Male	46 (61.3)
**Age at index reaction (years) Median (IQR)**	4 (4)
Age at DPT (years) Median (IQR)	6 (3)
**Time between Index reaction and DPT (months) Median (IQR)**	12 (32)
**Route of drug administration**	
Oral	74 (98.7)
Intravenous	1 (1.3)
**Duration of index reaction**	
≤ 1 h	20 (26.7)
> 1 h	55 (73.3)
**Treatment for index reaction**	
Antihistamine	42 (56)
Antihistamine + steroid	6 (8)
Adrenaline	3 (4)
No treatment	19 (25.3)
Unknown	5 (6.7)
**Number of reactions with the suspected drug**	
1	70 (93.3)
≥ 2	5 (6.7)
**Previous use of the suspected drug**	
Yes	54 (72)
No	20 (26.7)
Unknown	1 (1.3)
**Type of index reaction**	
Urticaria	23 (30.7)
Angioedema	3 (4)
Urticaria–Angioedema	4 (5.3)
Urticaria (not typical)	17 (22.7)
Anaphylaxis	3 (4)
Maculopapular exanthem	25 (33.3)
**Suspected drug**	
Amoxicillin-clavulanic acid	64 (85.3)
Amoxicillin	4 (5.4)
Phenoxymethylpenicillin (PenV)	5 (6.7)
Cefixime	1 (1.3)
Ampicillin-sulbactam	1 (1.3)
**PEN-FAST score**	
Median (IQR)	3 (1)
0	4 (5.4)
1	6 (8)
2	15 (20)
3	41 (54.6)
4	0 (0)
5	9 (12)
**Additional allergic disease**	
Atopic dermatitis	1 (1.3)
Allergic rhinitis	13 (17.3)
Asthma	5 (6.7)
Chronic urticarial	3 (4)
None	53 (70.7)
**Reaction to a suspected drug or any beta-lactam after DPT**	
No	68 (90.7)
Unknown	3 (4)
**Parental history of allergy**	
Yes	26 (34.7)
No	49 (65.3)

*IOR*, Interquartile range; *DPT*, Drug provocation test; *PEN-FAST*, penicillin allergy, five or fewer years ago, anaphylaxis/angioedema, severe cutaneous adverse reaction (SCAR), and treatment required for allergy episode. The major criteria included an allergy event occurring 5 or fewer years ago (2 points) and anaphylaxis/angioedema or SCAR (2 points); the minor criterion (1 point) was treatment required for an allergy episode. 0: very low risk,1–2: low risk, 3: moderate risk, 4–5: high risk

The median PEN-FAST score among patients was 3 (range: 0–5). When comparing patients with immediate versus delayed reactions, there were no significant differences in terms of age at the index reaction, age at provocation, the interval between the index reaction and provocation, recurrence of the same reaction, family history of atopy, eosinophil count and percentage, total IgE levels, or PEN-FAST scores (*p* > 0.05). However, a significant association was observed regarding sex. Immediate reactions were more common in female patients (*p* < 0.05).

There were no significant differences between sex groups and provocation results, parental history of allergy, age at index reaction, age at provocation, time interval between index reaction and provocation, recurrence of the same reaction, eosinophil count and percentage, total IgE levels, and PEN-FAST scores (*p* > 0.05).

**Fig. 1:**
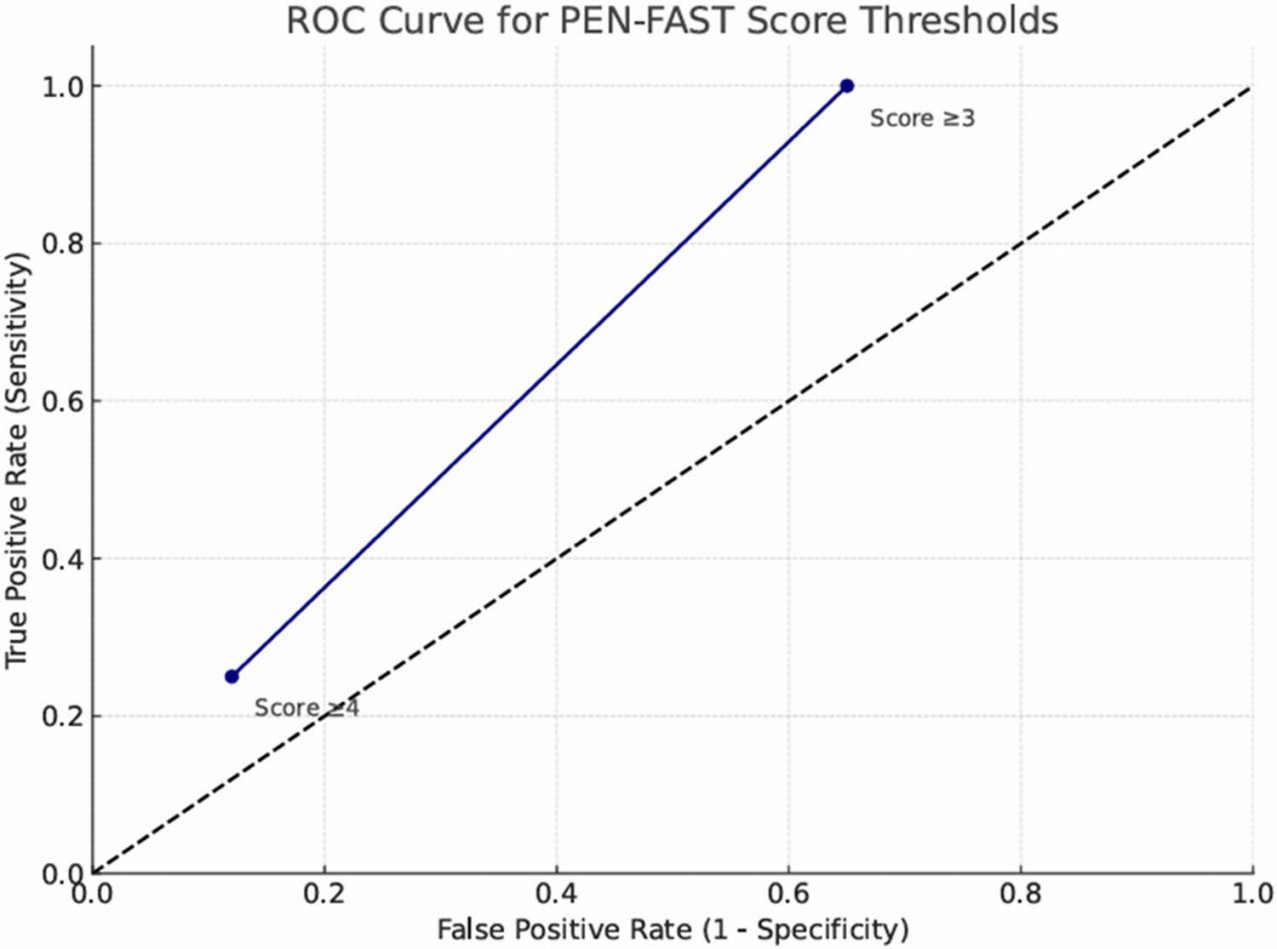
ROC Curve for PEN-FAST score

Upon evaluation of all cut-off points, the PEN-FAST scoring algorithm demonstrated a diagnostic performance with an area under the ROC curve (AUC) of 0.701 (95% CI, 0.59–0.90), indicating that it could correctly identify true drug allergy cases in approximately 70% of instances. These findings suggest that the test exhibits an acceptable level of discriminative ability (Fig. 1).

When a PEN-FAST score ≥ 3 was used as the cutoff, the sensitivity of the PEN-FAST test in identifying true drug allergy based on DPT results was calculated as 100% (95% CI 67,6–100), specificity as 35% (95% CI 33,8–53,7), positive predictive value (PPV) as 8% (95% CI 6,9–24,2), and negative predictive value as 100% (95% CI 91,2–100). When the cutoff was set at ≥ 4, sensitivity decreased (25% (95% CI 7,1–59,1)), whereas specificity and PPV increased (88% (95% CI 73,6–89) and 11% (95% CI 89,4–96,6), respectively).

The median age at provocation was 6 years (IQR: 3; range: 1–17). The median time interval between the last reaction and DPT was recorded as 12 months (IQR: 32; range: 2–264 months) (Table 1). In three patients with a history of anaphylaxis, DPT was performed twice (with penicillin and a penicillin + β-lactamase inhibitor combination), resulting in a total of 78 DPTs conducted. All three patients whose index reactions were classified as anaphylaxis exhibited cutaneous and respiratory system involvement. Each was treated with intramuscular adrenaline. The interval between the index reaction and the drug provocation test was at least one year, and all had a PEN-FAST score of 5 (Table 2).

**Table 2:** Characteristics of patients with index reaction anaphylaxis/DPT positive patients

Case No	Sex	Age (years)	Age at index reaction (years)	Drug at index reaction	Route of administration/location	Time to onset of reaction	Prior exposure to suspected drug before index reaction	History of reaction to suspected drug	Past reaction manifestations	Past treatment during reaction	PEN-FAST score	DPT
Characteristics of patients with index reaction anaphylaxis												
1	Female	5	1	Ampicillin-sulbactam	IV/Hospital	30 min	No	-	Generalized urticaria, wheezing	Adrenaline	5	Ampicillin (Negative) Ampicillin-sulbactam (Negative)
2	Male	9	2.5	Amoxicillin-clavulanic acid	Oral/Home	30 min	No	-	Urticaria angioedema, wheezing	Adrenaline	3	Amoxicillin (Negative) Amoxicillin-clavulanic acid (Negative)
3	Female	14	13	Amoxicillin-clavulanic acid	Oral/Home	1 h	Yes	+	Urticaria wheezing	Adrenaline	5	Amoxicillin (Negative) Amoxicillin-clavulanic acid (Positive) (urticaria 10 min after the second dose)
Clinical characteristics of DPT-positive patients												
1	Male	7	4	Amoxicillin-clavulanic acid	Oral/Home	2 h	No	+	Urticaria	No	3	Amoxicillin (Positive) (urticaria 30 min after the first dose)
2	Female	14	13	Amoxicillin-clavulanic acid	Oral/Home	1 h	Yes	+	Anaphylaxis	Adrenaline	5	Amoxicillin (Negative) Amoxicillin-clavulanic acid (Positive) (urticaria 10 min after the second dose)
3	Male	10	10	Amoxicillin	Oral/Home	8 h	Yes	+	Maculopapular exanthema	Antihistamines	3	Amoxicillin (Positive) (maculopapular exanthema 10 h after the first dose on day 2)
4	Female	5	5	Amoxicillin	Oral/Home	30 min	Yes	+	Urticaria	Antihistamines	3	Amoxicillin (Positive) (urticaria 10 min after the second dose on day 1)

*DPT, Drug provocation test; min: minute; h: hour*

**Fig. 2:**
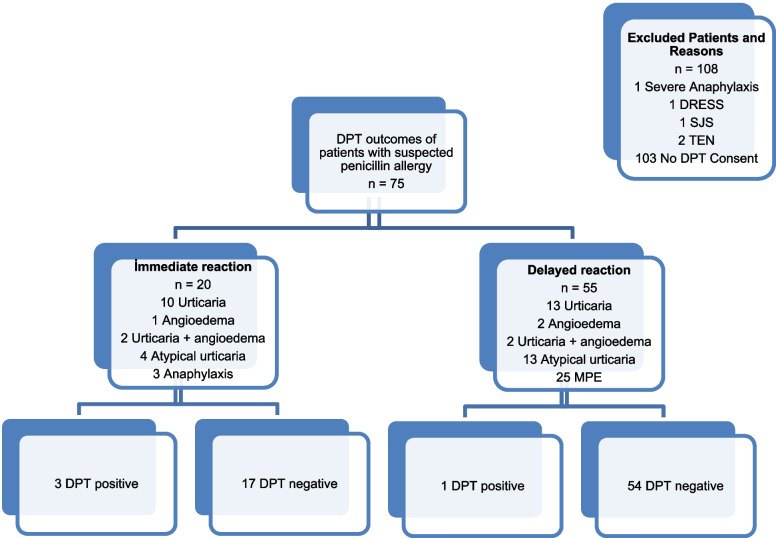
Reaction phenotypes and drug provocation test (DPT) outcomes/Excluded Patients and Reasons (SJS: Stevens Johnson syndrome TEN: Toxic Epidermal Necrolysis, DRESS: Drug reactions with eosinophilia and systemic symptoms, DPT: Drug Provacation Test)

Of the four patients with a positive DPT, two were male. Among the 20 patients with a history of immediate reactions, 3 (15%) had positive DPT results. In two cases, the reaction occurred with amoxicillin, and in one case with amoxicillin-clavulanic acid. In all three instances, urticaria developed during the DPT. A single dose of antihistamine was administered during the hospital observation period. Among the 55 patients with a history of delayed reactions, only one exhibited a positive DPT. A rash developed 10 h after the final dose on the second day of drug administration, and maculopapular exanthema was observed. Based on the positive DPT findings in these four patients, the use of penicillin group antibiotics was restricted. However, because none of the patients had a history of reactions to third-generation cephalosporins, they were informed that these agents could be used as an alternative BL group. Moreover, all reactions were limited to the skin, and no life-threatening allergic reactions (including anaphylaxis) were observed (Fig. 2) (Table 2).

When DPT-negative cases were re-evaluated after 3 months, 90.7% had not experienced any reaction to BL group antibiotics, although three patients (4%) opted not to use the drug despite the negative test result.

## Discussion

In the present study, the outcomes of DPTs were evaluated in children with suspected penicillin allergy presenting with both immediate and delayed reactions. In our series, DPTs were performed directly after the assessment of penicillin V-/penicillin G-specific IgE levels. DPT positivity was detected in only 5.3% of the cases. None of these patients experienced a severe allergic reaction.

Beta-lactam antibiotics—particularly penicillin—are frequently implicated in drug-related hypersensitivity reactions in children. In many instances, the reactions that occur are merely exanthems triggered by underlying infections, leading to patients being unnecessarily labeled as allergic to drugs. As observed in the present study, the confirmed diagnosis of a true drug allergy was very low, a finding that is consistent with previous studies [22–25].

Distinguishing between true drug allergies and infectious exanthems can sometimes be challenging [26]. Although immediate reactions are generally expected to occur within the first hour after drug administration, previous reports indicate that such reactions may be delayed by up to 6 h [11, 27]. In our cohort, urticaria was the most common manifestation among patients with immediate reactions, whereas maculopapular exanthema was most frequently observed in those with delayed reactions [13].

Furthermore, the present study identified challenges in obtaining accurate diagnostic information. Parents often were unable to fully articulate the nature of the reaction, and because of delayed presentation, critical details such as the reaction type, onset timing, the dose at which the reaction occurred, and the specific drug(s) administered were not reliably recalled. Notably, 54 patients (72%) had a history of similar reactions to the same drug. These findings underscore the importance of early evaluation following the initial reaction to prevent potential severe outcomes and to accurately differentiate true drug allergies from nonallergic exanthems.

In this assessment process, the PEN-FAST scoring system was also used in patients with suspected penicillin allergy [10]. In the pediatric population, Copaescu et al. reported an AUC value of 0.528 for the PEN-FAST score. Hanniet et al. found an overall AUC of 0.66 in both pediatric and adult populations, with an AUC of 0.71 in the adult subgroup. According to the literature, the discriminatory power of the PEN-FAST score appears to be significantly lower in children compared to adults. In our study, we observed an AUC of 0.71, which is consistent with values reported in adult populations and considered an acceptable level for this type of diagnostic tool [28, 29].

The median PEN-FAST score in our cohort was 3, classifying the group as moderate risk. When evaluating specific cut-off values, a threshold of 3 was found to be effective in ruling out beta-lactam allergy in patients with lower scores. However, among the three patients in our study with a history of anaphylaxis—all of whom had scores of 3 or higher—only one (with a score of 5) tested positive on drug provocation test. We believe this reflects the limited specificity of the PEN-FAST score in patients presenting with high-risk reaction histories. The notably low positive predictive value may be associated with the fact that many pediatric drug reactions are not true allergies. Future large-scale studies may help refine the parameters of the PEN-FAST score, enhancing its clinical utility in risk stratification and prediction.

In our study, although the unavailability of skin testing represents a limitation, it necessitated the use of specific IgE (spIgE) assays, which are routinely available in our laboratory. From a laboratory evaluation perspective, based on the data obtained in our study, we believe that our concerns regarding the necessity and clinical utility of this test are well-founded.

Skin and in vitro tests are commonly used in the diagnosis of BL allergy. However, in many allergy centers in Turkey, including our clinic, the recommended DAP Penicillin® (Diater Laboratories, Madrid, Spain) tests are not routinely available. When reviewing the studies conducted to date, a 2021 meta-analysis by Sousa-Pinto et al., which evaluated both adult and pediatric patients with a history of penicillin allergy, demonstrated that while skin tests have high specificity, their sensitivity in predicting hypersensitivity reactions was found to be below 50% [30]. In line with ongoing studies on beta-lactam antibiotics, particularly penicillins, Arıkoğlu et al. reported one year later that skin test protocols vary between centers and that sensitivity and specificity values are inconsistent [14]. A detailed evaluation of the literature reveals that several factors influence the findings related to skin testing. These include the often prolonged interval between the index reaction and the time of assessment, the limited data on the increasing use of various antibiotics over time, the availability of different penicillin preparations across centers, and variations in clinical practice. According to current literature, in children with a history of immediate BL allergy, skin testing followed by DPT is still recommended [31, 32]. In addition to this conventional approach, recent studies have proposed alternative strategies, suggesting that direct DPT may also be safe in children with a history of mild immediate reactions. [13].

In line with recent studies, our findings also emphasize that the rate of true hypersensitivity reactions among patients who underwent DPT was remarkably low. We were able to directly perform DPT—the gold standard diagnostic tool—without prior diagnostic testing in both immediate and delayed penicillin-related hypersensitivity reactions. Similarly, the literature reports that in both immediate and delayed reactions, except for severe cutaneous adverse reactions, direct DPT has been performed without preceding skin testing, and no life-threatening reactions were observed. [33–36]. In our study, patients with a history of severe anaphylaxis were excluded. Although this may limit the generalizability of our findings, it should be noted that severe drug-induced anaphylaxis is exceedingly rare in children. For instance, a study conducted by the International Rheumatic Fever Study Group reported an anaphylaxis rate of 0.2%—approximately 1 in 10,000 injections—among 1,790 patients receiving monthly benzathine penicillin G [37]. In the general population, the incidence of beta-lactam–induced anaphylaxis has been reported to range between 0.015% and 0.004% in other studies; however, there are only a limited number of studies focusing specifically on pediatric populations [38].

In our study, DPTs were positive in three out of twenty patients with a history of immediate reactions and in one patient with a delayed reaction. All reactions—both immediate and delayed—were limited to cutaneous symptoms, with no additional systemic manifestations. Considering these findings, in pediatric populations, it may be reasonable to consider omitting certain diagnostic steps, particularly in cases of mild, skin-limited immediate reactions such as urticaria, in order to minimize painful procedures for children. [39].

Furthermore, in cases where diagnostic tests confirmed hypersensitivity, families became more cautious regarding drug avoidance, which helped prevent subsequent reactions. Therefore, demonstrating the presence or absence of drug allergy appears to encourage families to adopt more prudent behavior. Notably, in the vast majority (90.7%) of patients in whom drug allergy was excluded by diagnostic tests, beta-lactam antibiotics were subsequently used without incident. By removing incorrect drug allergy labels, unnecessary drug avoidance could be prevented. Ultimately, this can contribute to both individual and public health while reaffirming the importance of diagnostic testing.

This study has several limitations. First, the relatively small sample size limits the generalizability of the findings. Additionally, the long interval between the index drug reaction and the drug provocation test in many patients may affect the reliability of both clinical and laboratory data, in addition to the patient-reported history. These factors should be taken into account when interpreting the results of our study.

In conclusion, our study demonstrates that DPTs can be performed by omitting the skin testing step in both immediate and delayed reactions. Similar to previous studies, we consider direct DPT to be safe in non-immediate reactions (excluding severe cutaneous adverse reactions—SCARs) and potentially applicable in mild, skin-limited immediate reactions. Except for severe non-cutaneous immediate reactions or in patients with a history of severe reactions such as anaphylaxis our approach, applied with a careful risk–benefit assessment, represents in children a pragmatic step toward accurate diagnosis under resource-limited conditions. We emphasize the need for this strategy to be supported by larger, multicenter studies. As is well known, drug reactions in this age group may often be misdiagnosed due to overlap with infection-related exanthems. Considering the increasing use of alternative antibiotics and the associated risk of resistance, the procedural difficulties children face during in vivo testing, and the implementation of the PEN-FAST scoring system, we believe our study offers a valuable perspective for both clinicians and patients.

## Data Availability

No datasets were generated or analysed during the current study.

## Abbreviations

BL: Beta-lactam
BLA: Beta-lactam allergy
DPT: Drug provocation test
IQR: Interquartile range
PPV: Positive predictive value
